# Management and outcome of vagus nerve stimulator implantation: experience of an otolaryngeal/neuropediatric cooperation

**DOI:** 10.1007/s00405-021-06943-x

**Published:** 2021-07-01

**Authors:** S. Grasl, S. Janik, A. Dressler, R. Diehm, G. Gröppel, K. Eichinger, M. C. Grasl, W. Gstoettner, M. Feucht, E. Vyskocil, W. D. Baumgartner

**Affiliations:** 1 Department of Otorhinolaryngology, Head and Neck Surgery, Medical University of Vienna, Vienna, Austria; 2 Department of Pediatrics and Adolescent Medicine, Division of Neonatology, Intensive Care Medicine and Neuropediatrics, Medical University of Vienna, Partner of the ERN EpiCARE, Vienna, Austria; 3 Department of Children and Adolescent Medicine, Hospital of St. John of God, Eisenstadt, Austria

**Keywords:** Vagus nerve stimulator (VNS) surgery, Children, Epilepsy, Outcome, Perioperative management

## Abstract

**Objective:**

Vagus nerve stimulator (VNS) implantation is an established therapy for pharmacoresistant epilepsy that is not amenable to curative epilepsy surgery. Historically, VNS implantation has been performed by neurosurgeons, but otolaryngologist involvement is increasingly common. In this retrospective study, we aimed to evaluate the efficacy and safety of VNS implantation in children and adolescents from the otolaryngologists’ perspective.

**Methods:**

This study included children and adolescents who had undergone VNS implantation at the study center between 2014 and 2018. Patient files were analyzed with regards to the durations of device implantation and hospitalization, postoperative complications, and clinical outcome, including seizure frequency, clinical global impression of improvement (CGI-I) score, and quality of life (QoL).

**Results:**

A total of 73 children underwent VNS surgery. The median age at implantation was 9.3 ± 4.6 years, and median epilepsy duration before VNS surgery was 6 ± 4 years. Lennox–Gastaut syndrome was the most common syndrome diagnosis (62.3%), and structural abnormalities (49.3%) the most frequent etiology. Operation times ranged from 30 to 200 min, and median postoperative hospitalization length was 2 ± 0.9 days. No complications occurred, except for four revisions and two explantations due to local infections (2.7%). Among our patients, 76.7% were responders (≥ 50% reduction in seizure frequency), 72.1% showed improved CGI-I scores, and 18.6–60.5% exhibited considerable improvements in the QoL categories energy, emotional health, and cognitive functions.

**Conclusion:**

Our results indicate that VNS implantation is a highly effective and safe treatment option for children and adolescents with AED-refractory epilepsies who are not candidates for curative epilepsy surgery.

**Supplementary Information:**

The online version contains supplementary material available at 10.1007/s00405-021-06943-x.

## Introduction

Epilepsy affects approximately 50 million people worldwide [1], one-fifth of whom are children under 15 years of age, who exhibit a wide-ranging clinical spectrum of this disease [2]. While most affected children are successfully treated with antiepileptic drugs (AEDs), 20–30% show pharmacoresistance, which is associated with high risks of intellectual disability, neuropsychiatric co-morbid disorders, increased mortality, and poor quality of life (QoL) [1, 3, 4]. Vagus nerve stimulation was approved for the treatment of AED-refractory epilepsy in the 1990s, and over 100,000 VNS devices have now been implanted worldwide. Vagus nerve stimulation is a well-established adjunctive therapy for epilepsy that is refractory to medical treatment but does not fulfil the criteria for respective/disconnective curative epilepsy surgery [5, 6]. Vagus nerve stimulation leads to persistent improvement of seizure control in ~ 50% of patients, with even better outcomes among children [4, 7–16].

Potential perioperative and postoperative complications of vagus nerve stimulation include intraoperative bradycardia and asystolia during lead impedance testing (0.01%), infections (3–8%), peritracheal hematoma, and nerve injury [17–26]. Later complications may be related to suboptimal vagal stimulation, recurrent laryngeal nerve co-stimulation, and/or mechanical stress—and can include delayed arrhythmias (bradycardia and asystolia), obstructive sleep apnea, and laryngopharyngeal dysfunction (dysphonia, cough, cervical pain, pharyngeal problems, and dyspnea). These complications occur in up to 66% of patients, but are often controllable by adjusting the stimulation parameters [6, 13, 17–26]. Drooling and hyperactivity have also occasionally been reported in children [14].

After primary VNS implantation, 4–16.8% of patients undergo revisions due to device malfunction, VNS therapy failure, intolerable side effects, or patient dissatisfaction [21–26]. The spectrum of these complications suggests that successful device implantation and positioning are critical. While VNS implantation has historically been performed by neurosurgeons [27], otolaryngologists are increasingly involved in this procedure due to their expertise regarding the surgical anatomy of the neck, and their far-reaching experience in the implantation of neuromodulation devices, such as the cochlear implants, and devices for hypoglossal nerve stimulation or and baroreflex modulation.

In the present study, we retrospectively evaluated various aspects of VNS therapy, to weigh the risks of device implantation against the possible benefits for seizure control and QoL.

## Materials and methods

We performed a retrospective study of all patients who had undergone a VNS implantation or a secondary VNS-related surgery at the Department of Otorhinolaryngology, Head and Neck Surgery of the Medical University of Vienna between March 2014 and December 2018.

### Data assessment

Data were obtained from the electronic epilepsy registry, which includes all patients who have undergone presurgical evaluation at the Department of Pediatrics and Adolescent Medicine since 2000. Patients’ records were retrospectively evaluated to retrieve sociodemographic data, epilepsy type, comorbidities, procedure, surgery length, immediate intraoperative and postoperative complications, duration and mode of postoperative care, vagus nerve stimulation-associated side effects, and AED treatment before VNS implantation and at last consultation.

### Preoperative period

To determine candidacy for epilepsy surgery, all patients underwent a standard-of-care presurgical evaluation at the MUW pediatric epilepsy center, which included long-term video-EEG-monitoring; neurological, neuroophthalmological, neuropsychological, and neuropsychiatric investigations; and MRI. Indications for implantation were discussed and decided at the MUW pediatric epilepsy interdisciplinary seizure conference.

### Surgical procedure

All VNS implantations were performed by the senior coauthors (W-D.B. and W.G.). The implantation procedure and technique was similar to that described by Reid et al. [28]. Supplementary Fig. S1 illustrates the most important steps of VNS surgery.

### Postoperative period

Following VNS implantation, the parameter settings were titrated on an outpatient basis by the treating epileptologists at the MUW pediatric epilepsy center (A.D., G.G., R.D., and M.F.). The patients’ caregivers were asked to keep seizure diaries—including seizure frequency, severity, and times of occurrence—before and after VNS implantation. The baseline parental seizure count of the primary seizure type prior to VNS implantation was compared with that at last observation. Patients showing a seizure frequency reduction of ≥ 50% were defined as “responders”.

QoL was assessed based on available patient records, and on standardized telephone interviews with caregivers, including evaluation of energy/activity level, concentration, alertness, memory, verbal communication, mood, performance at school, and development of life skills. QoL was categorized as better or much better since VNS, unchanged since VNS, worse or much worse since VNS, or unknown.

The Clinical Global Impression–Improvement scale (CGI-I) was used to assess whether a patient’s illness had improved or worsened compared to baseline before implantation. CGI-I scores were stratified as “much improved or very much improved”, “minimally improved”, “no change”, or “minimally worse or very much worse”. Postoperative pain was assessed using a visual numerical rating scale (vNRS), where 0 indicated no pain and 10 indicated maximal pain. This scoring was possible in 27 (37%) of 73 children, and was not applicable in the remaining children due to intellectual disability and limited communication.

### Statistical analysis

Statistical analyses were performed using SPSS version 26.0 software (IBM Corp. Armonk, NY, USA). Data are presented as median and mean ± standard deviation (SD) unless otherwise stated. Descriptive statistics were applied to evaluate the significance of demographic and clinical data, and were used for both subjective and objective quantitative measurable responses. The chi-squared test was used to compare nominal variables. We used the Pearson and Spearman correlations (correlation coefficient, *r*) to analyze linear relationships between metric and ranked variables, respectively. A *P* value of < 0.05 was considered statistically significant.

## Results

### Study cohort

Our analysis included 73 patients, comprising 32 girls (43.8%) and 41 boys (56.2%), with a median age of 9.3 ± 4.6 years (1.9–20.1 years). All children suffered from AED-resistant epilepsy. The median disease duration before VNS implantation was 6 ± 4 years (0.4–16.7 years). Patients had received a median of 6 ± 3.2 different AEDs (2–15 AEDs). Twelve children (16.4%) followed a ketogenic diet (KD). Prior to VNS implantation, six children (8.2%) had undergone resective epilepsy surgeries with unfavorable outcomes, including temporal lobe resection (*n* = 2), frontal lobe resection (*n* = 2),  callosotomy (*n* = 1), and hemispherotomy (*n* = 1).

The most common epilepsy syndrome was Lennox–Gastaut syndrome (*n* = 44; 60.3%), followed by West syndrome (WS) (*n* = 6; 8.2%), epileptic encephalopathy with continuous spike and wave during slow wave sleep (CWSWS) (*n* = 6; 8.2%), Dravet syndrome (DS) (*n* = 5; 6.8%), epilepsy with myoclonic-atonic seizures (MAE) (*n* = 5; 6.8%), epilepsy with myoclonic absences (*n* = 4; 5.4%), combined generalized and focal epilepsy (*n* = 2; 2.8%), and focal epilepsy (*n* = 1; 1.4%) (Table 1). The most common etiology was structural (*n* = 36; 49.3%), followed by combined genetic and structural (*n* = 16, 21.9%), and genetic (*n* = 11, 15.1%). MRI revealed epileptogenic lesions in 54 cases (74%). Table 1 presents the types of epilepsy and seizures, and electroencephalography (EEG) characteristics.

**Table 1:** Epilepsy and seizure characteristics of all children (*n* = 73)

		No. of patients (%)
Syndromes	Lennox–Gastaut syndrome West syndrome CSWS epilepsy Dravet syndrome Epilepsy with myoclonic-atonic seizures Epilepsy with myoclonic absences Combined generalized and focal epilepsy Focal epilepsy	44 (60.3) 6 (8.2) 6 (8.2) 5 (6.8) 5 (6.8) 4 (5.4) 2 (2.8) 1 (1.4)
Seizure types	Focal onset Generalized onset Unknown onset	38 (52.1) 34 (46.6) 1 (1.4)
Epilepsy types	Focal Generalized Combined: generalized & focal Unknown	38 (52.1) 22 (30.1) 12 (16.4) 1 (1.4)
Etiology	Structural Structural and genetical Unknown Genetic Infectious and structural Metabolic	36 (49.3) 16 (21.9) 11 (15.1) 6 (8.2) 3 (4.1) 1 (1.4)
EEG characteristics		
Brain function disorder	None Diffuse Focal Severe Missing	29 (39.7) 26 (35.6) 15 (20.5) 2 (2.7) 1 (1.4)
Cerebral excitability	None Focal Multifocal Generalized Missing	5 (6.8) 12 (16.4) 41 (56.2) 13 (17.8) 2 (2.7)
EEG pathology grade	I II III Missing	1 (1.4) 1 (1.4) 70 (95.9) 1 (1.4)

*CSWS* epileptic encephalopathy with continuous spike and wave during sleep, *EEG* electroencephalography

### Surgical procedure and perioperative period

A total of 54 primary VNS implantations were performed using Cyberonics 106 (*n* = 36), Cyberonics 103 (*n* = 9), Cyberonics 304 (*n* = 6), Sentiva 1000 (*n* = 2), and Cyberonics 303 (*n* = 1) devices, with no reported device failure. Of these 54 cases, 4 required generator replacement (all Cyberonics 106) during the observation period. Additionally, 15 generators that were implanted at other institutions between 2007 and 2014 had to be exchanged due to end of battery service, and were replaced by Cyberonics 103 (*n* = 4) or Cyberonics 106 generators (*n* = 11). Overall, the generators’ running time was 5.1 ± 1.6 years (2.5–7.7 years). Four complete system changes were performed due to electrode fractures. The primary implantations had been performed at other institutions between 2001 and 2013, with a median running time of 7.7 ± 3.4 years (4.9–14.1 years).

The median duration of surgery was 100 ± 17.1 min (70–165 min) for primary VNS implantation, 45 ± 12.1 min (30–60 min) for generator replacements, and 175.5 ± 54.3 min (85–200 min) for complete system change. The median impedance, measured during surgery, was 1600 ± 387.9Ω (980–3485 Ω). After VNS implantation, four cases (5.2%) required intensified observation at an intermediate care unit due to patients’ underlying diseases. In the remaining 73 (94.8%) interventions, the patients were transferred to the normal inpatient ward after a regular observation time in the recovery room. The median postoperative inpatient stay was 2 ± 0.9 days (0–5 days) with no significant differences between primary implantation, generator replacement, and complete system change.

Postoperative pain was managed using paracetamol alone (*n* = 29) or combined with metamizole or other non-steroidal anti-inflammatory drugs (NSAIDs) (*n* = 21). According to vNRS, the average pain level was 1 ± 0.9 (out of 10) (0–3). Perioperative antibiotic therapy was administered either as single-shot treatment or as perioperative and postoperative therapy over 9 ± 1.3 days (2–12 days). An amino-penicillin was used in 93.2% of patients.

### Complications and side effects

No severe complications (e.g., bleedings or asystolia) occurred during surgery. Dysphonia was noted in three cases (3.9%). One 2-year-old child suffering from WS exhibited a single self-limiting episode of postoperative bradycardia, which did not seem to be related to VNS implantation since the stimulation parameters of the VNS device had not been changed, and the bradycardia was self-limiting.

Two patients (2.7%) suffered an infection-associated incompatibility reaction against the implant, and were thus explanted and not re-implanted. In both children, the infection was localized at the generator area, and was first observed a month after surgery due to a local swelling fistulation and accompanying secretion. Three children died due to their underlying diseases.

### Seizure frequency and improvement after VNS implantation

**Fig. 1:**
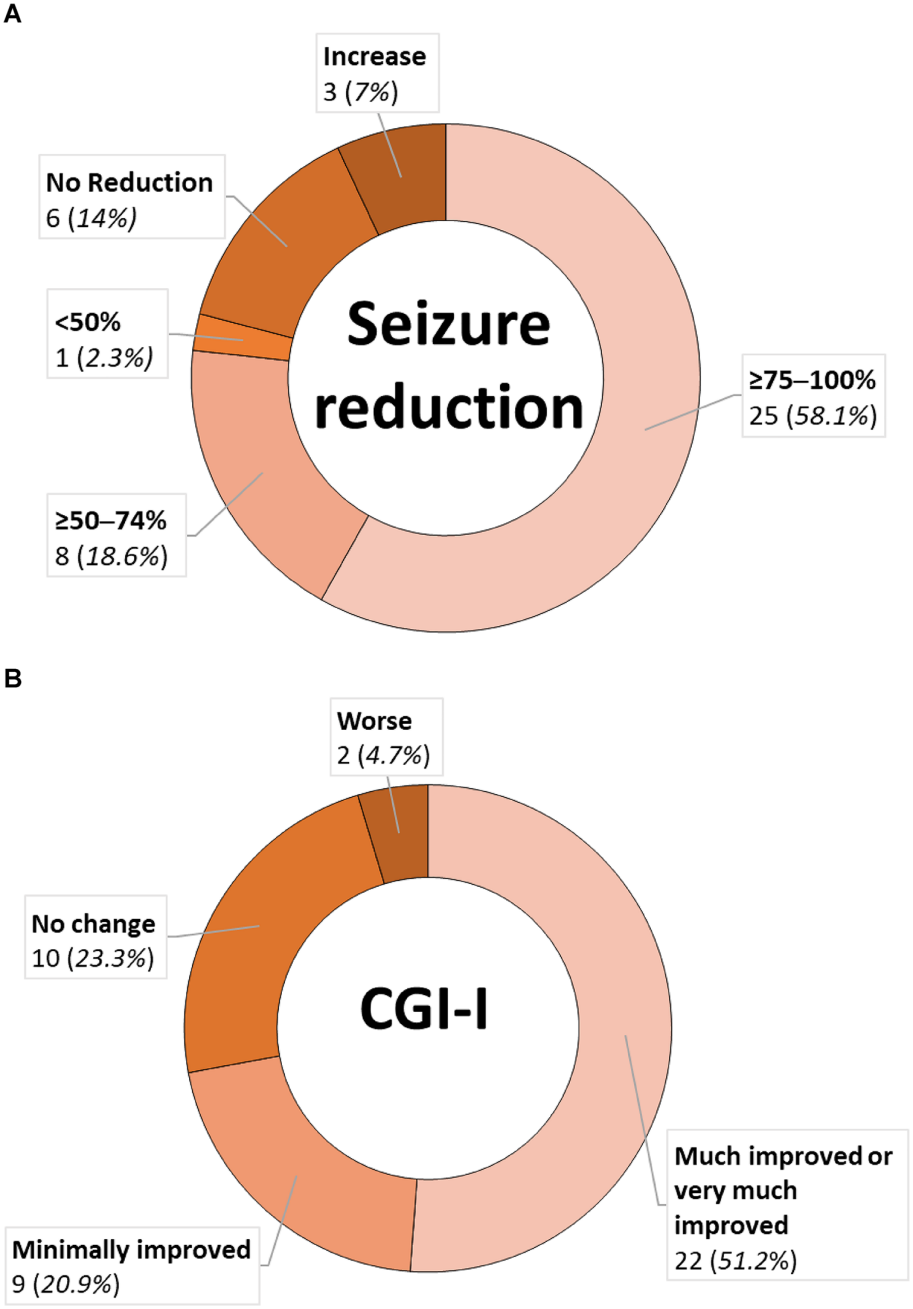
Reduction of seizure frequency **A** and results of the clinical global impression of improvement (CGI-I) assessment **B** after vagus nerve stimulation device implantation

Analyses for outcome assessment included only the patients with primary VNS implantation (n = 54). Six children were lost to follow-up, two required explantation, and three were deceased. Therefore, our analysis included follow-up data from 43 patients. The mean follow-up time was 3 ± 1.3 years (1–5.9 years). Among our patients, 76.7% were responders (n = 33), including 25 patients with a seizure frequency reduction of ≥ 75% after VNS implantation. No patient became seizure-free. One patient (2.3%) exhibited a frequency decrease of < 50%, six (14%) showed no improvement, and three (7%) showed an increased seizure frequency (Fig. 1A).

Etiology was a significant factor associated with therapy response. Patients with exclusively structural, unknown, or genetic etiologies showed a significantly higher therapy response than the remaining patients (*P* = 0.048). The various seizure syndromes were not associated with seizure outcome (*P* = 0.148). Seizure outcome was significantly correlated with age at seizure onset (*P* = 0.025; *r* =  − 0.363), with older age at onset associated with better response and seizure frequency reduction. Previous unsuccessful resective epilepsy surgery (*P* = 0.032) was also significantly associated with unfavorable seizure outcome in terms of seizure frequency. Seizure outcome was not correlated with sex, number of previously used AEDs, age at time of VNS implantation, duration of epilepsy prior to VNS surgery, or epilepsy-specific factors (e.g., EEG characteristics and types of seizure and epilepsy) (Table 2).

**Table 2:** Overview of factors correlating with objective and subjective therapy response after vagus nerve stimulator (VNS) implantation

	Responder		CGI-I	
	*P*	*r*	*P*	*r*
Age at seizure onset	0.025	− 0.363^a^	0.237	− 0.215^b^
Age at VNS surgery	0.491	0.108^a^	0.001	0.471 ^b^
Sex	0.769	–^c^	0.146	–^c^
Previous curative epilepsy surgeries	0.032	–^c^	0.228	–^c^
Duration of epilepsy before VNS	0.483	− 0.121^a^	0.023	0.407^b^
Number of AEDs before VNS	0.052	0.772^a^	0.261	0.182^b^
Epilepsy syndromes	0.147	–^c^	0.330	–^c^
Epilepsy types	0.073	–^c^	0.332	–^c^
Seizure types	0.279	–^c^	0.559	–^c^
Etiology	0.048	–^c^	0.013	–^c^
QoL				
Alertness	0.686	− 0.089^a^	0.003	0.443^b^
Concentration	0.637	− 0.104^a^	< 0.0001	0.569^b^
Energy	0.611	0.112^a^	0.001	0.486^b^
Memory	0.978	0.004^a^	0.986	0.003^b^
Mood	0.984	0.006^a^	< 0.0001	0.576^b^
Verbal communication	0.399	− 0.185^a^	0.029	0.334^b^
Progress at schoolwork	0.683	0.090^a^	0.037	0.319^b^
Development of life skills	0.236	0.257^a^	0.320	0.155^b^

*QoL * quality of life, AED antiepileptic drugs, *P*  *P* value, *r* = correlation coefficient

^a^Pearson correlation

^b^Spearman

^c^Chi-squared test correlation

### QoL and global impression of improvement after VNS implantation

Alertness increased in 60.5% (26/43) of the patients, and was the parameter that was most commonly improved by VNS therapy. We also found considerable amelioration of concentration (39.5%; 17/43), energy (41.9%; 18/43), mood (41.9%; 18/43), verbal communication (34.9%, 15/43), progress in schoolwork (39.5%; 17/43), memory (20.9%; 9/43), and development of life skills (18.6%; 8/43). After implantation, four patients (9.3%) exhibited reduced energy levels, and two exhibited aggravation of various aspects of QoL (Table 3).

**Table 3:** Quality of life (QoL) assessment after vagus nerve stimulator (VNS) implantation

	No. of patients (%)
Alertness	
Better or much better than before VNS	26 (60.5%)
Unchanged since VNS	14 (32.6%)
Worse or much worse than before VNS	1 (2.3%)
Not known	2 (4.7%)
Concentration	
Better or much better than before VNS	17 (39.5%)
Unchanged since VNS	22 (51.2%)
Worse or much worse than before VNS	1 (2.3%)
Not known	3 (7%)
Energy	
Better or much better than before VNS	18 (41.9%)
Unchanged since VNS	19 (44.2%)
Worse or much worse than before VNS	4 (9.3%)
Not known	2 (4.7%)
Memory	
Better or much better than before VNS	9 (20.9%)
Unchanged since VNS	15 (34.9%)
Worse or much worse than before VNS	1 (2.3%)
Not known	18 (41.9%)
Mood	
Better or much better than before VNS	18 (41.9%)
Unchanged since VNS	17 (39.5%)
Worse or much worse than before VNS	2 (4.7%)
Not known	6 (14%)
Verbal communication	
Better or much better than before VNS	15 (34.9%)
Unchanged since VNS	19 (44.1%)
Worse or much worse than before VNS	2 (4.7%)
Not known	7 (16.3%)
Progress with schoolwork	
Better or much better than before VNS	17 (39.5%)
Unchanged since VNS	15 (34.9%)
Worse or much worse than before VNS	0
Not known	11 (25.6%)
Development of life skills	
Better or much better than before VNS	8 (18.6%)
Unchanged since VNS	23 (53.5%)
Worse or much worse than before VNS	1 (2.3%)
Not known	11 (25.6%)

The CGI-I scores, based on the parents’ assessment of the patients’ overall condition, were significantly correlated with all QoL parameters, except memory and development of life skills (Table 2). According to the CGI-I scale, 51.2% (22/43) of the patients were “much improved” or “very much improved”, 20.9% (9/43) were “minimally improved” (Fig. 1B), and 28% exhibited no change or even a deterioration of the overall condition (4.7%). A positive CGI-I was significantly correlated with age at VNS surgery (*P* = 0.001; *r* = 0.471) and duration of epilepsy prior to VNS implantation (*P* = 0.023; *r* = 0.407) (Table 2). Children showing the highest benefit had a mean age of 8.9 years (SD: ± 4.3 years) and a mean epilepsy duration of 5.6 years (SD: ± 3.8 years), while children showing low/no improvement or deterioration of QoL had a mean age of 10.9 years (SD: ± 5.1 years) and a mean epilepsy duration of 8.5 years (SD: ± 3.7 years) (*P* = 0.022).

Etiology was also significantly associated with CGI-I improvement or deterioration. Seizure forms with a structural etiology exhibited significant improvement of CGI-I after VNS implantation (*P* = 0.029). Age at seizure onset, sex, previous anticonvulsive surgery or AED treatment, and other seizure characteristics were not associated with QoL parameters (Table 2). Finally, we examined the relationship between treatment response and QoL, and correlations between data from seizure responders and QoL responders. Seizure responders and non-responders did not differ in either CGI-I (*P* = 0.839, *r* = 0.037) or epilepsy-specific QoL after VNS implantation (Table 2).

## Discussion

In this retrospective study, we evaluated the short- and long-term effects and risks associated with VNS implantation in children and adolescents with pharmaco-resistant epilepsies. Little has been published about the increasing involvement of otolaryngologists in this surgical intervention and the immediate postoperative management. In the present study, surgical and perioperative care were provided by otolaryngologists, while neuropediatricians were responsible for the indication and the preoperative and postoperative care. This requires intense interdisciplinary communication and cooperation, especially during the postoperative phase when the first adjustments of VNS parameters are required.

Among our patients, the mean surgery duration varied from 30 to 200 min, depending on whether all components were implanted or only single components of the VNS had to be replaced which is in accordance with previously reported operation times [29, 30]. Our perisurgically measured mean impedance of 1580.9 Ω is also within previously reported limits [31]. Interestingly, the children did not consider the procedure itself to be very painful, with an average reported pain level of 1/10. Correspondingly, perioperative administration of paracetamol and/or NSAIDs was sufficient.

Concerns have been raised about patient safety due to reports of severe perioperative and postoperative complications, including major bleeding, cardiac arrhythmias, asystolia leading to cardiopulmonary resuscitation (CPR), and vocal cord paralysis [6, 13]. None of these severe events were reported in our patient group. Of the 73 children, 2 (2.8%) developed an incompatibility reaction against the implant, resulting in local infection of soft tissue, and requiring several revisions and ultimately explantation. These observations are in alignment with previously reported infection rates of ~ 5% [32–34]. The rarity of adverse effects was reflected by the short average hospital stay of only 2 days for all VNS surgery types. Most children were postoperatively transferred to the normal ward, with only a few cases requiring transition to an intermediate or intensive care unit. No persistent vocal cord dysmotility or paresis occurred. Transient stimulation-related dysphonia was observed in 3.9% of cases, and disappeared when parameter settings were changed (i.e., pulse width and strength).

With regards to the efficacy of vagus nerve stimulation for seizure reduction, 76.7% of our patients were “responders”, showing a seizure frequency reduction of ≥ 50%. Only 21% experienced no response or aggravation of seizure frequency. Previous reports show less favorable outcomes after VNS surgery [4, 35, 36]. Several aspects may have contributed to the success of VNS therapy in our patients. First, our pediatric collective was relatively young at VNS implantation, with a mean age of 10.4 years, and younger age was significantly correlated with better seizure outcomes. Several reports from other centers also show that VNS therapy is more efficient in children than adults [4, 35]. Additionally, the involved neuropediatricians had long-standing expertise in patient selection for VNS implantation, and subsequent medical assistance and treatment. Seizure control is a challenging and dynamic process [4]. Seizure frequency tends to increase with any infectious disease, and children may readily deteriorate. Moreover, 93% of our patients were still receiving AEDs at last follow-up, and it is difficult to disentangle the possible interactions between AED and vagus nerve stimulation with regards to seizure reduction.

Importantly, epilepsy treatment involves more than seizure counting. Reductions in seizure intensity and duration can have positive effects on the day-to-day lives of children with refractory epilepsy. This may explain the absence of a clear relationship between VNS-induced reduction in seizure frequency and improved QoL, as observed in the present study and in previous reports [16, 37].

In terms of QoL, we observed significant improvements in the parent-rated scores for energy and emotional health compared to preoperative assessments. Vagus nerve stimulation increases extracellular norepinephrine and serotonin availability in mood-regulating areas, e.g., the limbic system and prefrontal cortex. The mode of action resembles that of antidepressant medication, based on serotonin and norepinephrine reuptake inhibition [38, 39]. Positron emission tomography and single-photon emission computed tomography imaging studies in depressed patients demonstrate that vagus nerve stimulation changes the activity in the limbic system and prefrontal cortex [40, 41]. Regarding cognitive functions, we tested alertness, concentration, memory, and performance at schoolwork, and most patients showed considerable improvement after vagus nerve stimulation compared to baseline. Increasing evidence suggests that the stimulation may impact neuromodulatory systems, such as the locus coeruleus and the cholinergic systems, which modulate cognitive functions [42, 43]. Vagus nerve stimulation impacts different brain areas and neurotransmitter systems, with variation depending on the impulse frequency and magnitude [43].

Previously published work has mostly focused on single aspects of VNS surgery, therefore, our overall aim was to provide a comprehensive overview of the practical and holistic components of VNS surgery as seen from the point of head & neck. We describe the surgical process, consider the pre- and postoperative management including our favorable outcome. We also show the importance of efficient and patient centered multidisciplinary management, which is of upmost importance to ensure optimal patient selection and postoperative care from the experience of head & neck surgery. From the neuropediatric point of view, we believe that the presentation and comparison of both, objective and subjective outcome data after VNS surgery in a pediatric cohort, is of specific high value. To the best of our knowledge, this work up of VNS surgery is a quite unique approach. The major drawback of this study is its retrospective nature and an inherent bias due to the selection of information. Moreover, the heterogeneity of our patients, with a broad spectrum of different etiologies and epilepsy syndromes, limits the significance of predicting improvement after VNS implantation.

## Conclusion

Children and adolescents suffering from AED-refractory epilepsy greatly benefit from VNS therapy, substantiating the need to avoid delays in patient selection and VNS treatment. Otolaryngologist should be encouraged to apply their specific expertise in neck surgery towards the implantation of VNS devices, as it is an established surgical procedure that provides highly satisfactory outcomes in terms of safety and efficacy.

## Supplementary Information

Below is the link to the electronic supplementary material.

Supplementary file1 (DOCX 1356 KB)

## Data Availability

The datasets used and/or analyzed during the current study are available from the corresponding author on reasonable request.
