# Supplementary material for: Management and outcome of vagus nerve stimulator implantation: experience of an otolaryngeal/neuropediatric cooperation
Source: Eur Arch Otorhinolaryngol. 2021 Jul 1;278(10):3891–9. doi: 10.1007/s00405-021-06943-x (PMC8382619; doi:10.1007/s00405-021-06943-x)
Supplement: Supplementary file 1 — Supplementary file1 (DOCX 1356 KB) [file 405_2021_6943_MOESM1_ESM.docx]

| Supplemantary Fig. 1. Series of images showing the important steps of vagus nerve stimulator (VNS) implantation. | |
| --- | --- |
| **A** | **B** |
| 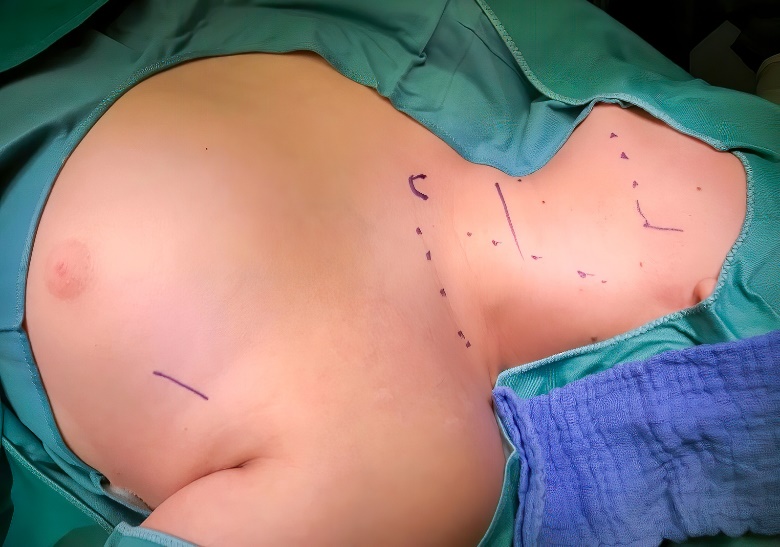 | **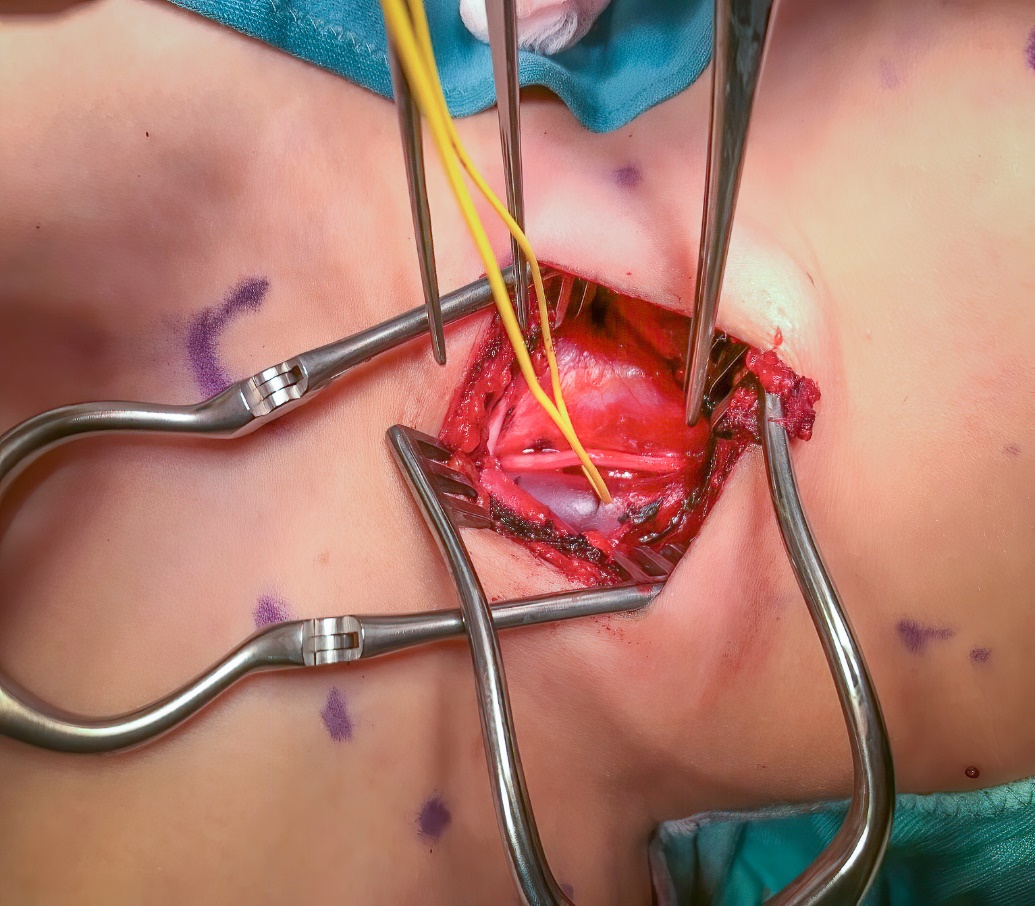** |
| **C** | **D** |
| 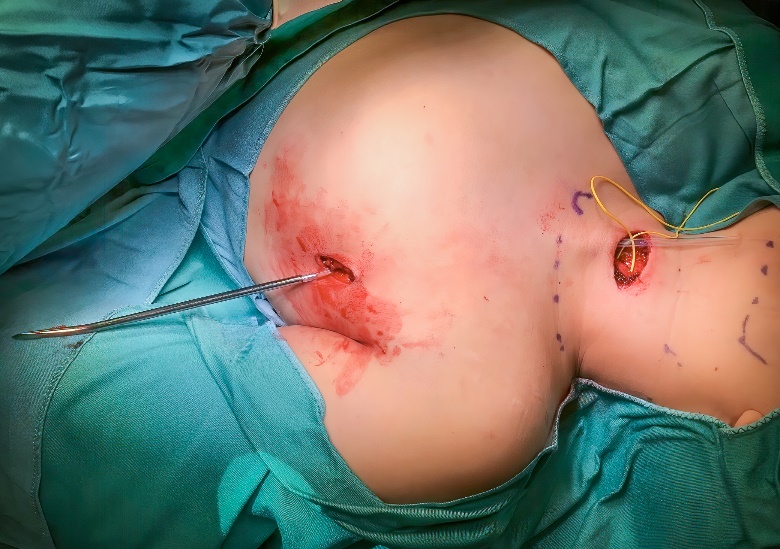 | 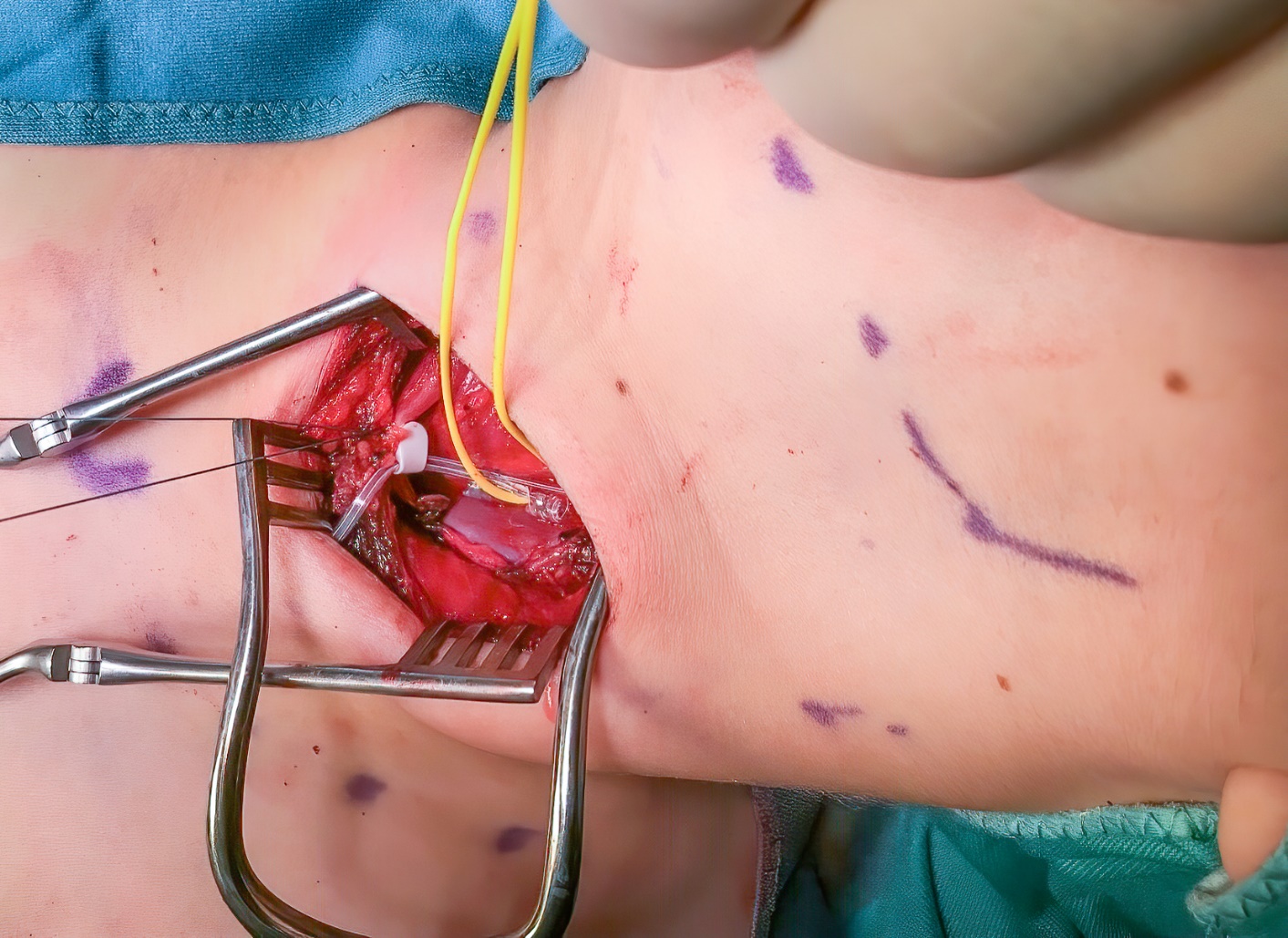 |
| **E** | **F** |
| 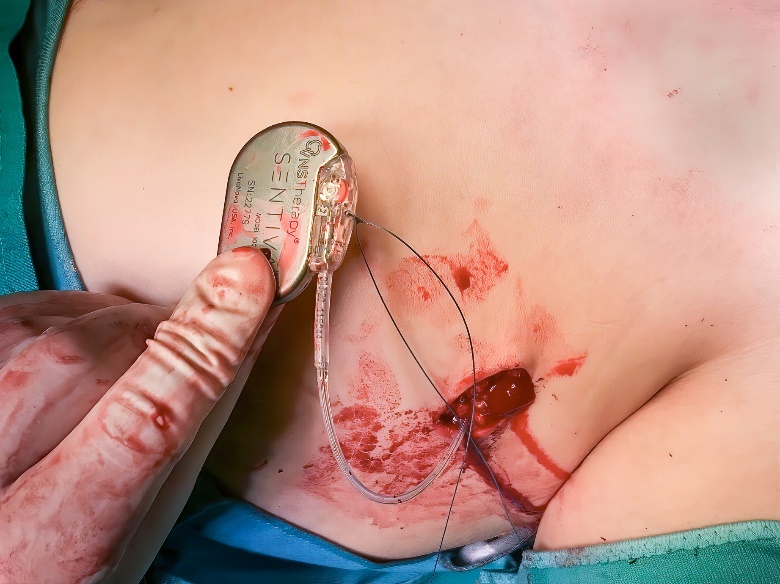 | 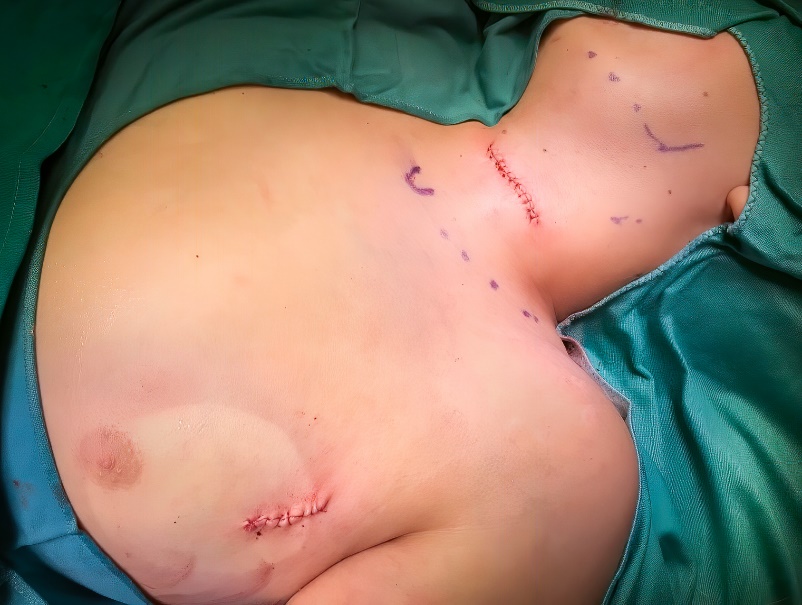 |
|  |  |
| Patients were subjected to orotracheal intubation and positioned for surgery, as shown in Figure 1A. All VNS implantations were left-sided. Anatomical landmarks were marked, and the skin was incised along a split line on the left side of the neck. After identification of the anterior border of the sternocleidomastoid muscle (SCM), the vagal nerve (Figure 1B) was dissected over a length of 6.5 cm, together with the internal jugular vein (IJV) and the internal/external and common carotid arteries (ICA/ECA/CCA). Next, a subcutaneous tunnel was formed using a 16-mm Redon’s stylus, stretching from the cranial of the clavicle to the left side of the chest. At the lateral border of the sternocleidomastoid muscle, a 4-cm incision was made to form a skin muscle pocket for the generator (Figure 1C). A Hartman forceps was used to place a pair of helical electrodes around the cervical vagal nerve. Three plastic plates were used to carefully fix these electrodes to the surrounding connective tissue, sternocleidomastoid muscle, and omohyoid muscle. Then the generator was inserted and coupled with the electrodes. If the VNS device was to be started intraoperatively, a low stimulation dose was set, after a final technical check with an impedance measurement and comparison between the measured and recorded heart rate. The generator was fixed using a non-resorbable 2.0 suture on the connective tissue of the pectoralis major muscle (Figure 1E), followed by multilayer wound closure (Figure 1F). | |
